# Inequitable access to healthcare in Africa: reconceptualising the “accountability for reasonableness framework” to reflect indigenous principles

**DOI:** 10.1186/s12939-021-01482-7

**Published:** 2021-06-13

**Authors:** Samuel J. Ujewe, Werdie C. van Staden

**Affiliations:** 1 Global Emerging Pathogens Treatment Consortium (GET-Africa), Lagos, Nigeria; 2 Centre for Ethics and Philosophy of Health Sciences, Faculty of Health Sciences, University of Pretoria, Pretoria, South Africa

**Keywords:** Health equity, Justice as fairness, Communal responsibility, Process, Solidarity, Sub-Saharan Africa, Healthcare context

## Abstract

**Background:**

The “Accountability for Reasonableness” (A4R) framework has been widely adopted in working towards equity in health for sub-Saharan Africa (SAA). Its suitability for equitable health policy in Africa hinges, at least in part, on its considerable successes in the United States and it being among the most comprehensive ethical approaches in addressing inequitable access to healthcare. Yet, the conceptual match is yet to be examined between A4R and communal responsibility as a common fundamental ethic in SAA.

**Methodology:**

A4R and its applications toward health equity in sub-Saharan Africa were conceptually examined by considering the WHO’s “3-by-5” and the REACT projects for their accounting for the communal responsibility ethic in pursuit of health equity.

**Results:**

Some of the challenges that these projects encountered may be ascribed to an incongruity between the underpinning ethical principle of A4R and the communitarian ethical principle dominant in sub-Saharan Africa. These are respectively the fair equality of opportunity principle derived from John Rawls’ theory, and the African communal responsibility principle.

**Conclusion:**

A health equity framework informed by the African communal responsibility principle should enhance suitability for SAA contexts, generating impetus from within Africa alongside the affordances of A4R.

## Introduction

This article takes issue with the suitability of Norman Daniels’ ethical approach to equity in healthcare, as summed up in the “Accountability for Reasonableness” (A4R) framework [20], towards effective policy reforms and equitable access to healthcare in sub-Saharan Africa. It argues that a more suitable equity framework of effective and substantive reach should engage with the ethic of communal responsibility dominant in sub-Saharan Africa. Daniels’ ethical approach is arguably the most utilised equity approach towards improving fairness or equitable access to healthcare in Sub-Saharan [61]. His approach has been largely adopted owing to, at least in part, its considerable use to inform healthcare reforms in the United States [14], and its adoption in the World Health Organisation (WHO) health improvement programs [20]. This has been taken up by adapting the framework towards equitable healthcare reforms in low-and-middle-income-countries [16, 18, 20–22, 66].

Specifically, the application of A4R in sub-Saharan Africa has met with various challenges. This raises concerns regarding the contextual limitations and suitability of the equity framework for Africa-specific healthcare reforms. Despite the numerous approaches being adopted by African countries towards equalizing healthcare, access to quality services remains significantly low or non-existent for much of the populations [61, 65]. This results in avoidable inequity in access to healthcare across population groups, as those who can afford private healthcare or health insurance get what they want, and those who cannot afford these do without even basic healthcare [1, 2, 49, 62, 67].

While the socio-economic dimensions of equity drives healthcare improvement initiatives [3, 31, 32, 35, 51], some health reform initiatives in the past two decades have been driven by the A4R framework, including the WHO “3-by-5” program and the REACT (Response to Accountable Priority-Setting for Trust in Health Systems) project in East Africa [8, 20]. Also, A4R has recently been adopted and applied in a clinical setting in South Africa towards improving equity in access to dialysis [46]. While these point to the potential value of A4R in sub-Saharan African contexts, it is important to note that these adaptations have so far not critiqued the foundational principles of A4R and seem to proceed with a priori assumption of its adaptability, given its prior success in the American context.

However, that an approach has been successful in some contexts does not always imply similar success in others. The Structural Adjustment Programs (SAPs), introduced in the 1980s, was peached to enhance healthcare equity in sub-Saharan Africa, but became grossly inadequate for the contexts. SAPs was proposed by high-income donor countries and designed by the International Monetary Fund (IMF) and the World Bank (WB) [38]. The package included trade liberalisation, currency devaluation, removal of government subsidies and price control, and cost recovery in healthcare and education. The introduction of SAPs led to a marked depression in health status marked by increased food insecurity and malnutrition, rising prevalence of ill health, and a decrease in access to healthcare in more than two-thirds of Sub-Saharan African (SAA) countries [12, 26, 34, 37, 50, 53].

Although SAPs was strategically designed to improve the conditions of poor countries, the negative effects, especially widening inequity in healthcare, shows that an externally generated strategy may not always be successful in navigating local conditions. The theorisation of SAPs may have been adequate and logically sound; yet its application in SAA was fraught with fatal consequences. The failure of SAPs is partly a function of discord between design and context – although a SAA initiative, was conceptualised and designed from outside of SAA contexts. Its application was marred by contextual limitations, leading to its failure. Adapting A4R towards enhancing healthcare equity in SAA contexts needs to be reassessed in terms of the conceptual harmony with indigenous SAA contexts.

For this reason, the aim of this article is to reconsider A4R for its applicability in SAA contexts and its accounting for the fundamental ethic, communal responsibility, that underpins and frames the pursuit of healthcare equity in Africa.

## Methodology

A4R and its applications towards health equity in sub-Saharan Africa are conceptually examined by considering the WHO’s “3-by-5” and the REACT projects for their accounting for the communal responsibility ethic in pursuit of health equity. We highlight the conceptual limitations of A4R that undermine its suitability for SAA contexts, despite its notable contributions to healthcare in the United States. We argue that an equity framework underpinned by African communitarian ethical principles may be more suitable, as it generates impetus from within the continent. Some of the challenges encountered in applying A4R in SAA may be ascribed to an incongruity between its underpinning ethical principle and the communitarian ethical principle dominant in the continent. These are, respectively: the *fair equality of opportunity* principle, and the *communal responsibility* principle. We do not aim to address questions of Western hegemony in bioethics, nor do we intend to belittle Daniels’ ingenious contribution. Rather, we revisit applications of A4R in SAA and provide reason to consider Africa-specific ethical approaches to address issues of healthcare equity. By positing communal responsibility, we do not attempt to dismiss the existence of individualism in SAA. Instead, we embrace communalism as one of the dominant principles that underlie social structures and individual existence across SAA.

Throughout this article, we use the term *African* in a similar sense as Godfrey Tangwa:

There is a great variety and diversity between the different African ethnicities, but they are all united by commonalities that give them a remarkable family resemblance analogous to the family resemblance of groupings that are in some ways remarkably different from one another but all justifiably bracketed under the term *Western*. ( [57], p. 104)

This recognises that while African countries and cultures are not homogenous, there are vast underlying commonalities that cut across them, including the broad understanding of personhood that is embedded in coexistence with others (see - [44, 64]). Since African countries are not homogenous, we deliberately did not attribute the communal orientation to countries, and even less as an exclusive orientation across all of Africa, but as a common orientation that pertains as a matter of degree among the people of Africa. Against this background, we use the term *community* to refer not only to locatable groups of people living in small villages across SAA and we also do not refer to the historical pre-colonial notion of community around Africa. Instead, *community* entails the concept of ‘cultural community’, where the natural sociability of the human being is recognised, and a sense of community is acknowledged as relevant to the total well-being and full realization of the human potential [29]. This would include both geographically locatable groups and groups of persons linked through societal structures, including governmental and non-governmental agencies.

## Results

### Accountability for reasonableness (A4R) and its application in SAA

A4R refers to the idea that the reasons or rationales for important limit-setting decisions to enhance equity in healthcare should be publicly available and accessible, and that these reasons must be ones that fair-minded people can agree are relevant for pursuing relevant care where resources are limited. It requires that the public not only have access to the decision made, but also the reasons underlying these decisions, and accept these as relevant towards providing high quality and equitable care for them [24]. The A4R framework is a practical tool that provides guidance to ensure fairness and equity in health decision-making processes. It aims to ensure that in a pluralist society, where reasonable disagreement about principles that should guide equitable health policy are likely, a fair process will help to establish acceptable decisions. This is laid out in four arms referred to as the four conditions, namely: publicity, relevance, appeal and revision, and regulative [20, 27].

#### Publicity condition

This requires that the rationale for decisions guiding the equitable distribution of healthcare resources or services be made publicly available, and accessible to those who may wish to raise objections or suggest alternative considerations [23]. It aims to ensure transparency in healthcare or policy decision processes, as “decisions regarding both direct and indirect limits to care and their rationales must be made publicly accessible” ( [24], p. 45). One basic principle is that similar cases should be treated similarly and that differential treatments must be justified by relevant reasons, the consistency of which will overtime convince people of the moral commitments of the institutions making them [24].

#### Relevance condition

It requires that the reasoning guiding decision-making for equity in healthcare and reforms are explainable to every reasonable person:

The rationales for priority-setting decisions should aim to provide a reasonable explanation of why the priorities selected were determined to be the best approach … a rationale is reasonable if it appeals to evidence, reasons, and principles accepted as relevant by fair-minded people. ( [27], p. 1576).

Under this condition, it should be possible for the concerned population to understand and accept the reasoning behind a particular health service fee, for instance. The kind of rationales provided should aim to provide a reasonable explanation on how any healthcare decision arrived at seeks to offer value for money. Is not sufficient to simply specify the goals, the goals must also appeal to reason, including values and principles that are acceptable to the concerned population [24].

#### Appeals and revision condition

This provides a mechanism for challenging and disputing decisions in healthcare, and provides opportunities for revision and improvement in the light of new evidence or questions [27]. It plays three distinctive roles:

- offering citizens a form of due process by which to attempt to revise adverse effects of inequitable healthcare decisions;
- giving those who are affected the opportunity to present their views towards improving decisions; and
- educating society about the need for setting limits for equity in healthcare through fair resource allocation decisions [20].

These hinge on the ground that healthcare decisions remain open to contestations to ensure that good arguments against the original decision can provided a fair route back into the policy decision process, and those affected by it can engage in the process [24].

#### Regulative condition

This requires the establishment of voluntary, public or legal strategies to ensure that the publicity, relevance and appeals conditions are met [20]. It affords the population a structure that ensures equity in healthcare, where their needs are prioritised in the decision-making process.

Thus, A4R constitutes a rational process that helps all stakeholders to understand the justification for (not) limiting certain services to some population groups, for instance, in order to enhance health equity. It ensures that policy decisions or interventions strategies are acceptable to all, at least in principle. The four conditions build on each other in determining the legitimacy of healthcare decision-making processes, so that satisfying all but one condition may not be sufficient in establishing health equity.

### Conceptual underpinnings of A4R

A4R hinges on a conceptual framework underpinned by the three moral questions for health, referred to as the *three focal questions* [20]:

- Is health of special moral importance?
- When are health inequalities unjust?
- How can we meet health needs fairly under resource constraints? [20]

The focal questions address three basic explanations underpinning A4R. An appropriate response to these questions will constitute an equity framework for healthcare and offer practical ethical guidance toward policy reforms [20]. Addressing the first focal question establishes the significance of health in ways that justify why societies should distribute healthcare resources more equitably than other social goods. The second question accounts for the many socially controllable factors, beside access to healthcare, that affect levels of population health and degrees of health equity within a system and between systems. And in addressing the third question, society stipulates the terms of fair process for making rationing decisions in distributing healthcare resources to the benefit of all [20].

Response to the focal questions provides a conceptual basis for pursuing justice in healthcare, identifies evidence of health inequalities that raise questions of justice, and offer pathways towards reforms for equity in healthcare. The framework helps society to examine the broader institutional setting and policies that mediate a population’s health, clarifying when a health inequality is unjust; what counts as a reasonable progress in reducing health inequality; and tests these in the context of actual policy choices. More importantly, it helps to develop a general account of fair process to legitimise decision-making processes [17].

Thus considered, the A4R approach presents great potential towards addressing equity questions surrounding the distribution of healthcare in Africa. It promises to inform substantive health system reforms, while legitimising the policy making process. It also establishes the obligation of health service providers to guarantee access, grant healthcare users the capacity to demand available services and hold policy makers accountable.

### The WHO’s 3-by-5 program

The WHO’s *3-by-5 program* was an initiative to scale up equitable distribution of anti-retroviral (ARVs) drugs and other HIV-related treatments, which set out to treat 3 million HIV/AIDS patients by the year 2005. Malawi, Tanzania, Ethiopia, and South Africa were some of the countries that featured in the project. Three key ethical guidelines of the *3-by-5 program* include: firm reference for public discussion; a process that is fair to all; and results that are ethically sound. The need for fair process in the *3-by-5 program* was considered essential for patient selection, given the lack of a specific principled approach at the time [19]. The A4R framework informs the four equity aspects of the program:

- Cost-recovery for drugs and services: Despite reasonable disagreements about what principles should guide the funding, the final decision hinged on the kind of reasons on which all could agree for the provision of free ARV treatments
- Medical eligibility criteria: Recommendation to consider best outcomes in the distribution of scarce medical resources, thereby extending the program to benefit the sickest patients
- Location of treatment facilities: Guided by the principle of fairness, the program aimed to balance the distribution of treatment facilities to cover a wide proportion of the population, as well as reach those most in need, and Priority to special groups: Guidelines were set to balance priority concerns between health workers who are most at risk of HIV infection and the sickest HIV/AIDS patients, and similar value disagreements [19, 20].

Considerations of fairness were observed in the decision-making process, Daniels notes, as several value disagreements were mitigated through the *fair deliberative process*. The process allowed room for counter cases to be raised, and allowed initial decisions to be revised in the light of further evidence [19]. Hence, the key aspects of the *3-by-5 program* reflect the four conditions of the A4R framework.

### The REACT project in Tanzania

Whereas the *3-by-5 program* was an externally developed initiative introduced to Africa, the REACT project was internally developed and deployed to enhance equity in access to healthcare among local populations. One key aspect of REACT was its adaptation of the A4R framework into health system reform processes in rural Tanzania [40, 41, 43, 47].

The project’s strategy involved describing existing policy practices in healthcare and supplementing them with the A4R framework to enhance effectiveness in the design and implementation processes. An evaluation of the REACT project showed that adapting A4R towards health system reform in Tanzania had wide appeal to both policy makers and the population for the following reasons:

- It involved multiple stakeholders to ensure that relevant values of affected communities were considered
- It informed the population about the rationale behind set priorities to create greater transparency, and enabled communities to know how healthcare resources were allocated, and
- It provided a mechanism for appeal to enable communities to express their dissatisfaction about some decisions [40, 41, 43].

### Challenges encountered in Africa

While the A4R framework has been widely accepted and implemented in various health policy processes in the United States [14, 17, 20–24, 48], it also attracted interest in healthcare reforms and intervention in Africa. Notable among these are is use in the: WHO “3-by-5” project [19, 20, 39, 66]; and REACT project [8, 40–43, 47]. The 3-by-5 and REACT projects were reviewed for the conceptual analysis in this article as they represent some of the most extensive and sustained attempts to implement A4R in practice towards achieving health equity in SAA. While the 3-by-5 program was a regional initiative that applied the A4R approach in various central and southern African countries [61], REACT was a country-based initiative that adopted A4R use in various locations within one African country, Tanzania. Analysing these two project side by side thus provides both broad and focused outlooks on the attempts to implement A4R in African settings. Both of these projects adopted the A4R framework as the equity platform on which to base targeted distribution of healthcare for specific population groups.

The *3-by-5 program* was widely acclaimed for enhancing a speedy scale-up of ART in Sub-Saharan Africa. For instance, the program was shown to benefit large numbers of rural populations in Malawi, and was recommended for uptake across Africa [25, 36]. A rapid scale-up was also recorded in Ethiopia [4]. However, several challenges were noted in the scale-up programs in different countries. In Ethiopia, it was acknowledged that HIV prevention and management of chronic care patients still posed a major concern, as the health system had yet to adopt a chronic care model linking care delivery with community-and-home-based interventions for HIV/AIDS patients [4]. In Malawi, about three quarters of those who started treatment defaulted, with half confirmed to have died shortly afterwards [68]. The most prevalent reason for defaulting was the cost of transportation from home to clinic, in spite of many patients being traceable. Van Damme et al. [63] attribute the problem to an existent incapacity, coupled with the additional burden of scaling-up. They note, for instance, that the healthcare sector was largely under-resourced and lacking in personnel and facilities for basic healthcare; hence including the ART roll-out program into routine healthcare posed additional challenges.

Like the A4R framework, the ethical principle underlying the *3-by-5* program focuses on individuals’ benefits, without recourse to the relevant socio-ethical contexts in which healthcare is situated. Thus, the *3-by-5* program was ethically misaligned with the African contexts in which it was implemented. A viable ethical approach to equity in access would account for individuals’ interests no less, but also recognise the central role of their networks of relationships and responsibilities.

In applying A4R in the REACT project, specific limitations were observed for local conditions in Tanzania. Among these, Maluka [42, 43] notes the need:

- for more engagement with affected communities in the decision-making process than the framework suggests
- to recognize underlying power asymmetries between affected communities and policy makers, and
- to recognize the nature of the local context’s socio-cultural traditions.

Additionally, Mshana et al. [47] observed that:

- the ethical approach was considered by local communities as being too technical and complicated
- many potential stakeholders might not have had the knowledge, skills or experience to effectively contribute to the process, which made some participants feel intimidated, and
- the analytical description of relevant reasons was complex and difficult for policy makers to communicate to the relevant population.

Overall, there was inadequate understanding of the process and its mechanism for influencing legitimacy and fairness in the local Tanzanian context, as reflected in health service management processes and outcomes [42]. The first set of limitations noted in both Maluka et al. [42] and Mshana et al. [47] show the need to engage local meanings within the people’s socio-ethical context; the second present the challenges of adopting a procedural approach that is unfamiliar to the local context; and the third presents socio-cultural challenges, as A4R does not account for local socio-cultural factors relevant to healthcare. If A4R was perceived as too technical or complex by local populations, it may not literally mean that the people simply lacked knowledge. Rather, it shows that the knowledge system underpinning A4R is different from that in the local context.

Additionally, three baseline studies evaluating the limitations of A4R in Tanzania, Kenya and Zambia found that while the involvement of communities and other stakeholders feature prominently in official policies, actual decision-making processes at district levels had substantial shortcomings in terms of participation, and the four conditions of A4R were not ensured in practice in any of the three studies [7, 40, 41, 69] – indicating marked gaps in the application of the A4R framework at all levels [8]. Likewise, findings from an action research in these three countries indicate that although the A4R principles were acknowledged as relevant and accommodated into existing set of organisational values, there was a great challenge and difficulty in grasping and applying the concept of A4R in daily practice [8].

Our position is that this challenge and difficulty hinge on substantive value differences between the modes of inquiry and analysis of the local contexts and those underpinning the A4R framework. The difficulty can be ascribed to an incongruity between the underpinning principles of A4R and the dominant ethical principles in African contexts. There is need for “… a broader and more detailed analysis of … the socio-cultural context … illuminating areas that require special attention to promote sustainability” ( [42], p. 14).

## Discussion

### Incongruity between underpinning principles of A4R and African ethics

It is relatively easy to attribute the challenges to A4R in African settings to technical difficulty in understanding or applying the framework. It is important to note, however, that contextual value differences and substantive variances in the principles underpinning A4R and those dominant in African ethical contexts pose a major challenge. Primarily, A4R is founded on the ethical principle of *Fair Equality of Opportunity*, while the principle of *Communal Responsibility* predominates in African ethical contexts. A more effective framework for equitable allocation of healthcare in African contexts, we contend, should crucially account for the principle of communal responsibility.

### A4R and the fair equality of opportunity principle

The A4R approach is premised on a functional relationship between preserving health and the kind of opportunities open to an individual in any given society. While a variety of opportunities open to individuals to pursue may depend on key economic and cultural features, and socialisation and/or historical developments of their particular societies, their physiological functioning is significant in determining the kind of opportunities they can actually pursue [15]:

Impairment of normal functioning through disease and disability restricts an individual’s opportunity relative to that portion of the normal range his skills and talents would have made available to him were he healthy … disease and disability shrinks his share from what is fair. ( [15], pp. 33–34)

This claim is informed by John Rawls’ theory of justice as fairness, which prescribes the principle of *fair equality of opportunity (FEO)* as essential for equity in the distribution of social goods [20]. FEO is concerned with the principle that free and rational individuals in any society, each of whom are concerned to advance their own interests, would accept in an original position of equality as stipulating their basic terms of agreement [52]. Specifically:

- each person would have the same indefensible claim to a fully adequate scheme of equal basic liberties, which is compatible with the same scheme of liberty for all; and
- social and economic inequalities must be attached to offices and positions open to all, and should be of the greatest benefit to the least-advantaged members of society [52].

A4R hinges on *b* above. FEO appeals to A4R given its potential to defend the obligation towards equity in access to healthcare. For instance, FEO would insist on fair advantage to those who are economically, socially or circumstantially less advantaged in the distribution of healthcare resources [20]. In other words, allowing greater access to healthcare to those who are worse off would reduce their relative social disadvantage and offer them a chance to pursue their desired and available opportunities within their society.

Considering its formulation, the FEO principle is more suited to societies that emphasise individual liberty over and above networks of social relationships. This may be typical of some societies in North America or Western Europe but is far from what predominates in most African societies. In African contexts, communitarian relationships are emphasized alongside the individual’s place. African ethical contexts hinge on a principle of communal responsibility.

### African ethic of communal responsibility

The moral outlook of SAA societies is constituted by the attribute of responsibility towards communities and individuals, which serves the welfare of both. This is in contrast to the attributes of individual opportunity with underlying right claims underpinning A4R. It is worth noting that the communal responsibility approach we ascribe to SAA is not an exclusive phenomenon in any or all nations so considered. It is not an orientation that necessarily applies to each person in Africa but is more relative and perhaps especially “vertically”, on the ground so to speak. Also, not all African countries or communities are homogenous in their approach to communal perspectives. Some countries subscribe to this principle more than others, and we acknowledge this relative difference while referring to the underlying dominant principle of communal responsibility.

Gyekye [30] articulates the difference between the ethic of individual rights and the ethic of communal responsibility. He notes that concern for human well-being constitutes the hub of the African axiological wheel, where this sociality or relationality prescribes an ethic of responsibility. This essentially involves individuals in social and moral roles in the form of obligations and commitments to other members of their community. In this context, the ethical values of compassion, solidarity, reciprocity, cooperation, interdependence and social well-being constitute the moral principles that impose duties on individuals with respect to the community and its members [30].

This ethics outlook differs from the rights ethic underlying A4R. Where individuals’ rights are emphasised, there are no immediate moral obligations and commitments to others simply in virtue of belonging to the same community. An individual is obligated to another only insofar as the latter can make viable claims that commit the former. Conversely, the ethic of communal responsibility does not eliminate benefits due to individuals. Individuals may lay claims to their entitlements but must always be conscious of their corresponding responsibilities to others in their web of relationships. For instance, among many cultural groups in Nigeria, male or older children are entitled to larger shares of family inheritance. However, the inheritance comes with a corresponding share of responsibility towards bearing the family’s burdens, including taking care of the widowed mother or wife; and the financial, educational, and other needs of younger/dependent siblings. Thus, the right to a larger share of inheritance is recognised, but always with a corresponding responsibility for the family.

By a Western account, it may be easy to assume that individuals’ rights are obliterated, undermined or trumped by the kind of responsibility enjoined in African moral spheres. It is worth noting, however, that:

The communitarian ethic acknowledges the importance of individual rights, but it does not do so to the detriment of responsibilities that individual members have or ought to have toward the community or other members of the community … the communitarian moral theory considers responsibility as an important principle of morality …. ( [29], p. 66)

African contexts present a moral sphere of rights interwoven with responsibilities. Here, claiming some rights does not relieve persons of relevant responsibilities, either to themselves or to their network of relationships. An African approach to equity in access to healthcare would be defined not only by the right to access services, for instance; rather, emphasis would be placed on the relevant communities’ (e.g., government agencies) responsibilities towards restoring the health and well-being of affected individual members. It would crucially subsist in balancing such rights with essential responsibilities. Emphasis on communal wellbeing does not and should not imply a neglect of individuals’ interests. The priority is a dual responsibility in sustaining the health and wellbeing of every member, and the community as a whole.

The communal responsibility principle implies that interdependency is acknowledged in the pursuit of equitable access in African healthcare [61]. It is considered morally reprehensible in African contexts, for instance, for persons to live economically superfluous lives, while their family (siblings or parents) live in abject poverty. Healthcare equity, by this principle, would seek to balance the well-being of individuals with that of others in their network of relationships.

### The incongruity

The ethical underpinning of A4R hinges on the ideal of individual opportunity to provide justification for the obligations to healthcare equity. However, African ethics contexts are different, as described above. This incongruity implies that A4R is in principle misaligned to African healthcare contexts, as exemplified in the REACT case. A viable equity framework must supplant or address the conceptual limitations of A4R. It must also be premised on the ethic of communal responsibility, not solely fair equality of opportunity. This points to the significance of contextual differences and acknowledgement of the challenges they pose. While some moral principles may hold good universally and timelessly, their application in particular concrete situations must not dispense with local perspectives and contexts [55].

Adaptations of A4R to African contexts of healthcare have thus far overlooked these contextual differences, insisting on its viability as a universal ethical framework. Yet, the mark of a universally adaptable framework:

… is that it provides a clear principle of action that is sensitive to both moral agency and moral patients and that it plastically applies equally to all global communities and societies without necessarily attempting to make uniform particular rules of action or foist the particular or peculiar moral dilemmas, quandaries, obsessions and preoccupations of some on all … We need always to keep in mind the context and perspective … of particular actions or procedures. ( [55], p. 158)

The REACT and 3-by-5 programs do not account for the ethic of communal responsibility underpinning local contexts of healthcare, thereby limiting their successes. Potential claims of A4R’s universal applicability are thus weakened. There is need for an equity framework that accounts for the local communitarian contexts of African healthcare.

Developing a framework that aligns with local socio-ethical contexts is crucial for optimal uptake and success for equity in access to healthcare. African contexts and approaches must take centre stage in developing the relevant framework. This would engender greater success in the formulation and execution of initiatives towards equitable access to healthcare. For healthcare initiatives to be effective, efficient, and fair, they must be integrated at several interconnected levels: between persons seeking and delivering healthcare, within the social network of each person, and across the trajectory of each person’s life [10]. For instance, it is not enough to develop a mechanism that provides free drugs to HIV/AIDS patients without considering their social, cultural and economic contexts, and those of their networks of relationship. To be equitable and effective, healthcare programs must be informed by these varying factors.

### Pathway for an African equity framework

In light of the above, it is crucial to map out a relevant equity framework for African contexts or reconceptualise A4R to reflect the African ethics contexts in which it is adapted. While developing the alternative approach is beyond the scope of this paper, we propose initial outlook for a viable pathway to healthcare equity in Africa. First, the framework should take seriously the ethical, social and cultural dimensions of African contexts. This cannot be overemphasised, as health and healthcare challenges are encountered within social and cultural communities. The approach must hinge on the principle of communal responsibility underpinning African ethics. Also, it would be informed by local knowledge systems to ensure effective communication and uptake among stakeholders in the decision-making process. Consideration should be given to specific social and cultural factors that were not anticipated in the original formulation of  A4R.

Second, the A4R approach hinges on Western analytic tradition in mainly employing the Rawlsian approach of “fair equality of opportunity”. The equity framework for African healthcare would incorporate the ethic of communal responsibility, which has a different methodological approach. An analytical approach may not be equally useful when applied in contexts that appeal to other forms of problem-solving methodologies:

It is a question whether all problems that face us … can be solved by a purely analytical method …. The analytic paradigm of knowledge … is not the only one. There are other types of human knowledge … much better developed and more prominent in non-Western cultures … there are aspects of reality and human life and existence with which that [analytic] paradigm … cannot adequately deal with. ( [56], pp. 227–228)

Drawing from an African methodological perspective is important, as the knowledge components can make significant contributions towards addressing Africa-specific problems [33]. It is imperative that an African approach is explored in developing an equity framework for healthcare in Africa. The framework will be underpinned by an African method of ethical analysis. This would involve emphasising people-centred processes, as reflected in the African ethic of communal responsibility.

Third, the African healthcare equity framework will employ an even-handed approach, accounting for varied socio-cultural factors while keeping universal phenomena in perspective. If we insist on a strictly African ethical approach, without recourse to the universal, we could lose some benefits of other useful healthcare reform initiatives:

There can be no culturally and psychologically perspective ethics without taking account of the diversity of moral lives, but there can be no ethics at all without universals, allowing a means of trying to stand aside from particulars to make meaningful ethical assessments. ( [9], p. 38)

Thus, the framework would consider African modes of moral explanations without dismissing the fact of its existence within a moral universe. Accordingly, the framework could draw from relevant universal attributes of A4R, but at the same time emphasise the African moral outlook.

Fourth, the African ethics framework would incorporate key attributes of the ethic of responsibility: solidarity, process, reciprocity, and harmony:

- The solidarity attribute entails a process of self-understanding through others [5, 28, 45, 54, 58, 61] and individuals’ personal and social responsibilities and commitments are always informed by the wellbeing of the whole community. The framework will be informed by the local population’s socio-cultural understanding of moral responsibility.
- The attribute of process emphasises the idea of becoming and existing through others [6, 44, 54, 60, 61, 64]. It indicates the vital social interconnectedness through which obligations and commitments arise. The framework will reflect this societal interconnectedness in its formulation and execution.
- The attribute of reciprocity is reflected by essential interdependencies emphasised in African settings [11, 13, 61]. Communitarian interdependency means that the ill-health of one person invariably bears on the health of another. The framework will not only consider the health and wellbeing of individuals, but of whole communities.
- The harmony attribute will ensure that the framework looks to the wellbeing of communities and individuals. Holistic restoration is of the utmost concern in African understandings of health and healthcare [61]. Given that harmony is ascribed to as the greatest good in African moral thought [59], restoration of individuals and communities will underpin the African healthcare equity framework.

## Conclusion

Although A4R has provided considerable guidance toward healthcare equity in Sub-Saharan Africa, a more suitable framework of effective and substantive reach should engage with and be driven by the ethic of communal responsibility dominant in sub-Saharan Africa. This ethic does not feature in A4R. Past experiences with the WHO’s *3-by-5* program and the REACT project underscore the contextual limitations and shortcomings of A4R and the need for developing an Africa-specific healthcare equity framework. Such a framework holds promise of generating impetus from within Africa towards equity in access to healthcare, drawing on the continent’s strengths, alongside the affordances of A4R. The wide socio-cultural diversity in Africa means that the relevant framework must be flexible enough to allow for specific adaptations in different contexts. Some adjustments may be needed to match subtleties in the varying cultures. Nonetheless, much untapped potential lies in the development of an equity framework guided by the African ethic of communal responsibility.

## Data Availability

NA
